# Some biological properties of spiny eel (*Mastacembelus mastacembelus*, Banks & Solander, 1794) living in the Upper Euphrates River Basin, Turkey

**DOI:** 10.1038/s41598-021-91223-1

**Published:** 2021-06-03

**Authors:** Mehmet Zülfü Çoban, Mücahit Eroğlu, Mustafa Düşükcan

**Affiliations:** 1 Keban Vocational Schools, Firat University, Keban, Elazığ, Turkey; 2 Faculty of Fisheries, Firat University, 23119 Elazığ, Turkey

**Keywords:** Ecology, Zoology, Limnology

## Abstract

This study was carried out to determine some bioecological characteristics of *Mastacembelus mastacembelus*, which is the only species of Mastacembelidae family living in Turkey. Fish samples were caught between 2014–2018 from Keban Dam Lake, one of the most important reservoirs of the upper Euphrates Basin. In totally, 348 *Mastacembelus mastacembelus* individuals were examined, including 178 males and 170 females. The age distributions were defined between the I–XV age groups. Total lengths ranged from 14.20 to 81.80 cm in males and from 15.60 to 77.30 cm in females. Total length–weight relationships were calculated as W = 0.0083 × TL^2.6516^ for males, W = 0.0043 × TL^2.8310^ for females and W = 0.0063 × TL^2.7256^ for all population, and the growth type was estimated as “negative allometric”. The von Bertalanffy growth parameters for all individuals were computed as L∞ = 90.99, k = 0.13, t_0_ = − 0.45. The total (Z), natural (M), fishing (F) mortality rates and exploitation rate (E) were estimated as Z = 0.313, M = 0.270, F = 0.043 and E = 0.137, respectively. The length at first capture (Lc) was found as 50.72. The optimum, maximum and economic yields were calculated as E_0.5_ = 0.361; E_max_ = 0.776; E_0.1_ = 0.664, respectively.

## Introduction

Mastacembelids or spiny eels (Teleostei: Synbranchiformes: Mastacembelidae) are a freshwater fish family. The family encompassing Mastacembelus (61 species), Macrognathus (24 species) and Sinobdella (1 species) genus includes 86 species^1^. However, the Mastacembelidae family is distributed in the Middle East, in Southeast Asia and north of China, most of its species live in Africa. Members of this family that are anguilliform fishes are mostly river forms^2–5^.

The only species of Mastacembelidae family in Turkey is *Mastacembelus mastacembelus* and is distributed in Tigris, Euphrates and Asi River systems^6,7^. The dorsal, caudal, and anal fins of the *M. mastacembelus* individuals living in Africa are jointed, but in individuals living in Turkey are not integrated. They live in lotic and lentic systems where are muddy and sandy grounds which are usually plentiful in vegetation and low in elevation. They hide in plants or bury in bottom muds to protect themselves during daytime^5,8,9^.

**Figure 1:**
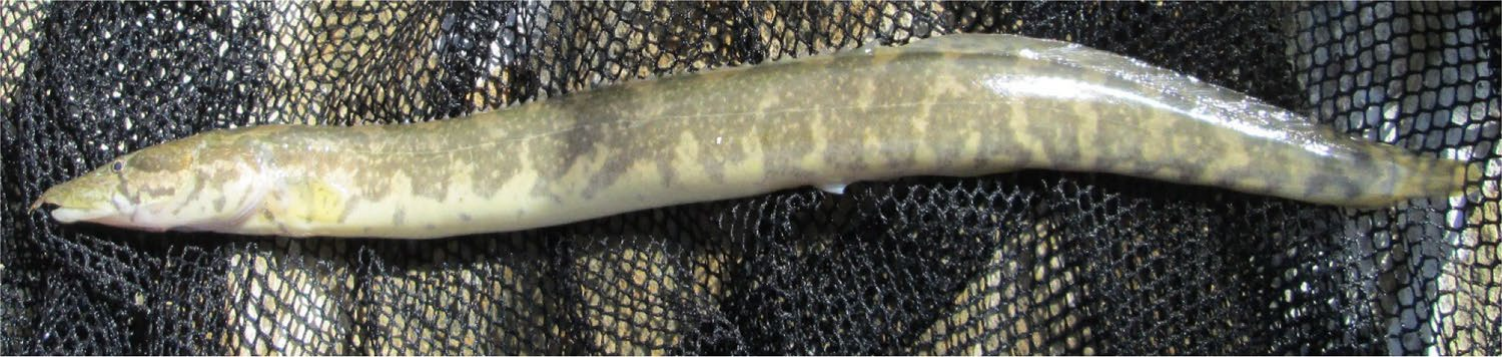
A sample of *M. mastacembelus* caught during the study (original photo).

Some of the morphological features of *M. mastacembelus*, which is called Mesopotamian spiny eel, are as follows: Dorsal spine 33–35; Anal spine 3–3; Dorsal fin ray 70–78; Anal fin ray 70–78; Caudal fin ray 16–21; Pectoral fin ray 18–21; Abdominal vertebrae 36–37; Caudal vertebrae 49–51; Total vertebrae 85–88; Lower jaw length 0.7–1.9; Upper jaw length 1.3–3.2; Head depth 3.3–6.1; Body depth 6.7–11.2; Rostral length 1.6–3.1 (Fig. 1)^10,11^.

Several studies have been carried out on the morphological characteristics^5,10,11^, embryo development^12^, digestive system content^13^, otolith size-fish length^14^, fish age-otolith size^15^, reproduction and growth characteristics^16–18^ of *M. mastacembelus* throughout Turkey. This study has got importance for being the first scientific research on the growth parameters of *M. mastacembelus* distributed in the Keban Reservoir located on the Euphrates River. Also, no studies were found on the mortality and exploitation rates, the length at first capture (Lc), the length at recruitment (Lr) and yield per recruit (Y/R) of *M. mastacembelus*.

## Materials and methods

### Study area and sample collection

**Figure 2:**
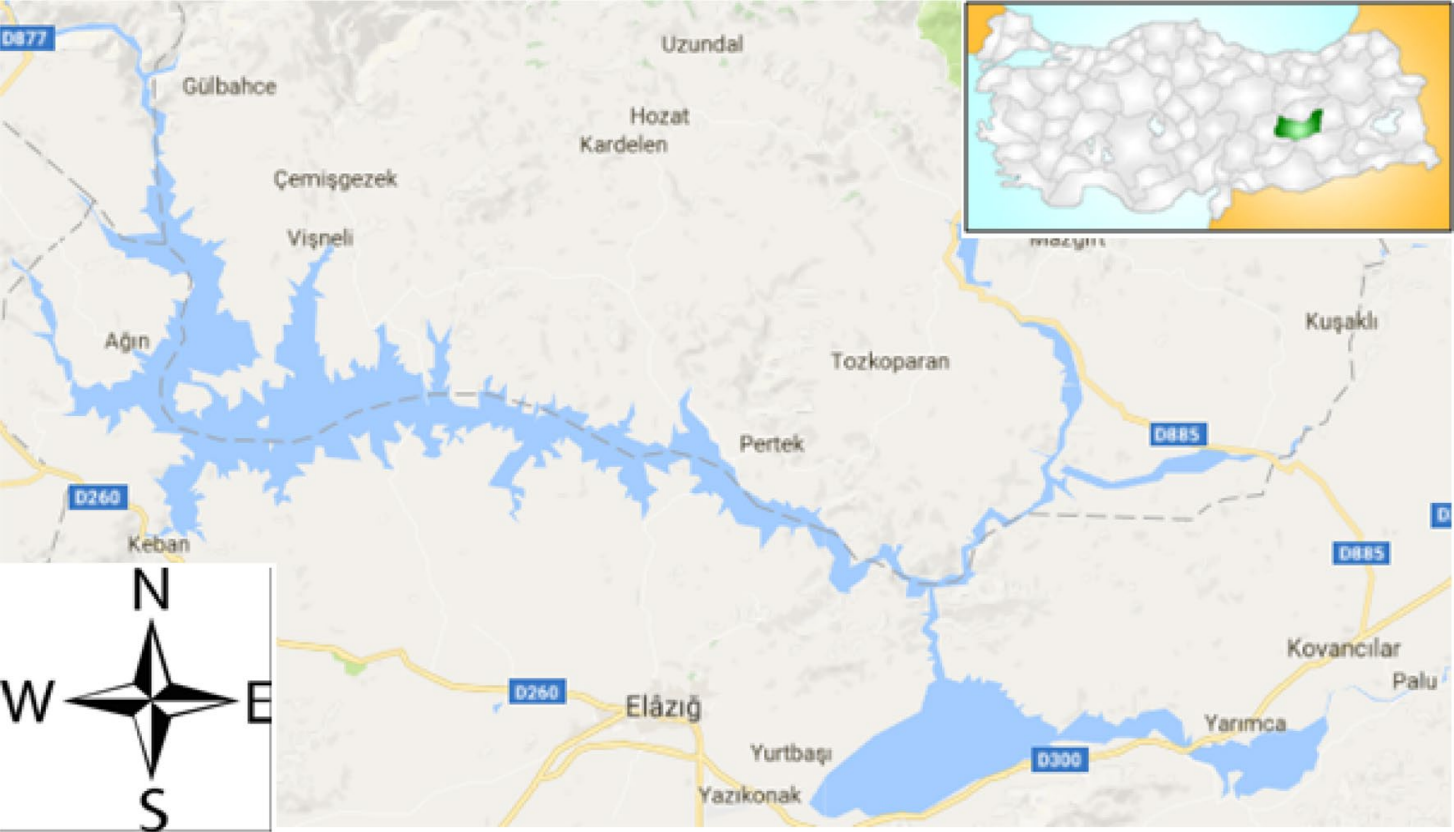
The map of Keban Dam Lake [Google Maps: https://www.google.com/maps/@38.8025012,38.9170508,9z (Accessed 10 February 2021)]^21^.

Keban Dam was built between 1965 and 1975 in the Keban district for electric production and irrigation. It is the second larger artificial lake in Turkey, was built on Euphrates River in the eastern part of Turkey (within 38° 37′ and 39° 20′N; 38° 15′ and 39° 52′E). The dam lake is 845 m above the sea level and has 675 km^2^ surface areas at maximum level, 160 m in maximum depth and 64,100 km^2^ in basin area (Fig. 2)^19,20^.

This study was carried out between 2014 and 2018 in Keban Dam Lake. Fish samples were caught using gill nets with 22, 28, 34, 36, 42, and 55 mm mesh sizes and crayfish fyke-nets, which used prevalently in the region, with D form and 36 mm stretched mesh size, structured with five hoops and a barrier. Most of the samples were taken from crayfish fishermen during crayfish hunting periods (December–June)^18^. Total length (TL, cm) and weight (W, g) of all fish samples were measured and its sexes were noted according to Lagler et al.^22^.

### Age and growth

Ages of samples were counted by using vertebrae. Because Gümüş et al. examined various bony structures in *M. mastacembelus* and reported that vertebrae are the most suitable structures for age estimation of this species. The vertebrae were immersed in boiling water for approximately 5 min. The vertebrae were then cleaned with a soft cloth and washed with alcohol. Larger vertebrae samples were processed with decolourant for about 1 min and immersed again with water. The vertebrae were dried at 105 °C for 15 min to increase the visibility of annular patterns, and they were examined in alcohol using a trinocular microscope (Olympus CX41 microscope and Olympus DP25 monitoring system), magnification of 10 × and 15 ×^18^.

The sex ratio of the samples was investigated using the Chi-Square test (*X*^*2*^)^23^. Condition factor (CF) was determined from CF = (W × 100)/L^3^ equation^24^. Length–weight relationships were computed from the Le Cren’s equation and the investigation of the age-length relationship of the *M. mastacembelus*, the von Bertalanffy growth equations (VBGE) were used^25^. The growth performance of fishes was estimated with Munro’s growth performance (phi-prime) index (φ′)^26^. Equations:

$$\begin{gathered} {\text{W}} = {\text{ a}} \times {\text{TL}}^{{\text{b}}} \hfill \\ {\text{L}}_{{\text{t}}} = {\text{ L}}\infty \times \left[ {1 - {\text{e}}^{{( - {\text{K}} \times ({\text{t}} - {\text{t}}_{0} ))}} } \right] \hfill \\ {\text{W}}_{{\text{t}}} = {\text{ W}}\infty \times \left[ {1 - {\text{e}}^{{( - {\text{K}} \times ({\text{t}} - {\text{t}}_{0} ))}} } \right]^{{\text{b}}} \hfill \\ \varphi^{\prime} \, = {\text{LogK}} + 2{\text{LogL}}\infty \hfill \\ \end{gathered}$$

where: L_t_: length of the fish at age t; L∞: asymptotic length; K: brody growth coefficient; t_0_: age of the fish at 0 cm length; W_t_: weight of the fish at age t; W∞: asymptotic weight; a and b: constants of the length–weight relationship^25^.

The VBGE parameters (L∞, K and t_0_) and Standard Error’s (SE) were calculated from the age-length data by using the FAO-ICLARM FiSAT II package^27^. Length–weight relationships of samples and standard error (SE) of a and b values were determined with SPSS 21.0 statistical software (SPSS Inc.). The confidence interval (CI_(%95)_) values of VBGE parameters (L∞, K and t_0_) and a and b values in the length–weight relationships were calculated with CI = SE × t_(n−1)_ equation (t_n−1_ is the critical value of the theoretical t-distribution for n − 1 degrees of freedom)^25^. The differences between the length–weight relationship parameters and VBGE parameters of male and female individuals were examined by ANCOVA test^23^.

### Mortality

The total mortality rate (Z) was estimated with the linearized catch curve based on age composition data. In this method, a linear regression analysis was performed for x = age, y = ln(N). In this regression, the slope (b) is accepted as “Z”. The natural mortality coefficient M was calculated by Pauly’s empirical formula (lnM = − 0.0152–0.279 × ln L∞ + 0.6543 × ln K + 0.4630 × lnT), while the fishing mortality coefficient F was computed by subtracting M from Z (F = Z − M). The exploitation ratio E was calculated by the formula E = F/Z^25^.

### The length at first capture (Lc)

The mid-point of the smallest length classes in the catch during the study period was accepted as the recruitment length (Lr). The length at first capture (Lc) was determined graphically by the cumulative catch curve analysis according to Pauly^24^.

The corresponding age at first capture (tc) was calculated as:

$$t_{(Lc)} = t_{0} - \frac{1}{K} \times LN\left( {1 - \frac{Lc}{{L\infty }}} \right)$$

### The yield per recruit

The yield per recruit of *M. mastacembelus*, caught in this study, was calculated using the following model of Beverton and Holt.

$$\frac{Y}{R} = F \times \exp \left[ { - M \times (tc - tr)} \right] \times W\infty \times \left[ {\frac{1}{Z} - \frac{3S}{{Z + K}} + \frac{{3S^{2} }}{Z + 2K} - \frac{{S^{3} }}{Z + 3K}} \right]$$

where: Y/R is the yield per recruit, F is the fishing mortality, M is the natural mortality, t_c_ is the age at first capture, t_r_ is the age at recruitment, W∞ is the theoretical maximum weight, Z is total mortality, K is growth coefficient and S equal to following equation: ${\text{S}} = {\text{e}}^{{{-}{\text{K}} \times ({\text{t}}_{{\text{c}}} - {\text{t}}_{0} )}}$^25^.

The growth type of fishes, the differences between total lengths, weights and condition factors of males and females were assigned by Student’s *t* test using SPSS 21.0 Computer Program.

## Results

### Age and sex distribution

178 (51.15%) of a total of 348 *M. mastacembelus* individuals were male and 170 (48.85%) of them are female. It was determined that the ages of investigated individuals ranged between the I–XV age groups. The overall sex ratio was estimated as 1:1.03 (females/males), this result was not statistically different from the 1:1 value (*X*^2^ = 0.09 < *X*^2^_(1, 0.05)_ = 3.84, p > 0.05).

### Length and weight distribution

**Figure 3:**
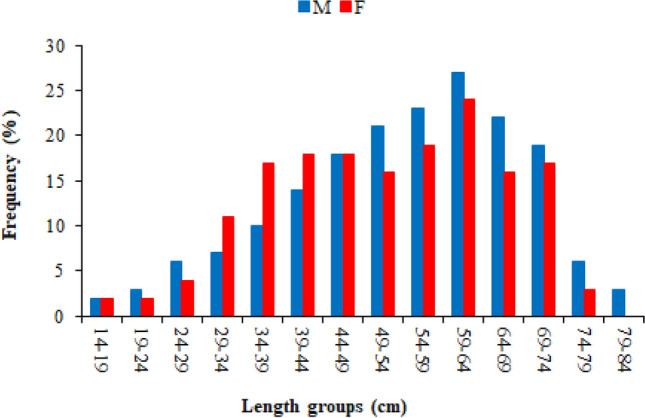
The length distribution of *M. mastacembelus* population inhabiting Keban Dam Lake.

Total length values ranged from 14.20 to 81.80 cm in males and from 15.60 to 77.30 cm in females. Total length measurements of males and females were statistically divergent in VII, VIII, IX and X age groups (p < 0.05) (Table 1). In the length frequency distribution, the maximum number of fishes were detected in 59–64 cm length group; with 8.46% (27 individuals) for males and with 7.52% (24 individuals) for females (Fig. 3).

**Table 1:** Total length (cm) and weight (g) values *of M. mastacembelus* population inhabiting Keban Dam Lake.

Age groups	$\overline{x}$ ± S.h. (min–max)						*T* test for lengths	*T* test for weights
	Male			Female				
	N	Total length	Weight	N	Total length	Weight		
I	4	17.60 ± 1.60 (14.20–20.80)	15.80 ± 1.77 (13.20–21.00)	2	15.90 ± 0.30 (15.60–16.20)	13.60 ± 0.40 (13.20–14.00)	p > 0.05	p > 0.05
II	8	25.58 ± 1.17 (24.00–30.20)	44.72 ± 2.61 (41.00–55.00)	12	25.76 ± 1.36 (19.20–29.70)	46.69 ± 6.48 (25.00–70.10)	p > 0.05	p > 0.05
III	11	36.39 ± 2.01 (27.20–45.70)	114.09 ± 15.80 (58.00–192.40)	16	32.57 ± 0.70 (27.00–38.40)	86.90 ± 6.90 (51.80–162.80)	p > 0.05	p > 0.05
IV	18	39.58 ± 1.48 (28.70–50.10)	151.19 ± 16.47 (58.00–301.90)	24	37.09 ± 1.53 (29.10–49.60)	127.84 ± 16.56 (50.40–295.50)	p > 0.05	p > 0.05
V	22	46.75 ± 1.17 (41.20–59.60)	251.10 ± 24.42 (150.00–576.30)	22	45.23 ± 1.81 (31.20–58.40)	241.51 ± 31.57 (64.00–593.00)	p > 0.05	p > 0.05
VI	25	55.38 ± 1.09 (47.10–65.40)	342.94 ± 19.54 (228.00–584.00)	24	50.97 ± 2.25 (36.70–64.60)	311.28 ± 32.97 (94.00–534.00)	p > 0.05	p > 0.05
VII	27	60.83 ± 0.63 (57.10–69.10)	438.79 ± 13.80 (344.50–594.90)	31	56.14 ± 1.70 (41.80–64.70)	415.55 ± 32.29 (130.70–608.00)	p < 0.05	p > 0.05
VIII	19	65.41 ± 0.57 (62.40–70.00)	541.04 ± 17.41 (442.00–673.80)	14	62.11 ± 1.53 (48.10–66.70)	536.51 ± 37.23 (190.30–632.00)	p < 0.05	p > 0.05
IX	12	68.78 ± 0.58 (66.50–73.10)	637.83 ± 25.39 (502.00–787.40)	9	64.28 ± 1.69 (53.80–68.40)	583.16 ± 23.90 (484.00–671.51)	p < 0.05	p > 0.05
X	11	69.72 ± 0.24 (68.10–71.10)	671.22 ± 25.07 (554.00–826.00)	5	66.14 ± 1.40 (63.00–70.20)	612.09 ± 48.28 (485.00–760.00)	p < 0.05	p > 0.05
XI	6	72.08 ± 0.49 (69.90–73.00)	641.03 ± 33.71 (553.10–774.00)	4	69.35 ± 1.81 (64.00–72.00)	732.30 ± 88.91 (488.20–886.00)	p > 0.05	p > 0.05
XII	6	73.55 ± 0.68 (72.20–76.00)	815.33 ± 50.76 (670.70–982.90)	3	72.33 ± 0.64 (71.30–73.50)	772.40 ± 8.43 (758.00–787.20)	p > 0.05	p > 0.05
XIII	4	75.48 ± 1.37 (72.10–78.20)	786.10 ± 94.53 (600.00–980.00)	2	74.15 ± 1.25 (72.90–75.40)	847.00 ± 39.00 (808.00–886.00)	p > 0.05	p > 0.05
XIV	3	76.87 ± 2.07 (72.90–79.90)	782.80 ± 97.95 (589.30–906.00)	2	77.05 ± 0.25 (76.80–77.30)	984.57 ± 25.73 (958.84–1010.30)	p > 0.05	p > 0.05
XV	2	80.90 ± 0.90 (80.00–81.80)	980.60 ± 150.50 (830.10–1131.10)	–	–	–	–	–

The weight measurements varied from 13.20 to 1131.10 g for males, and from 13.20 to 1010.30 g for females. According to the *t* test results, the weight measurements of males and females were not found statistically different in all age groups (p > 0.05) (Table 1).

### Length–weight relationships

As a result of the covariance analysis (ANCOVA) applied to the length–weight relationship of male and female individuals, it was determined that there was no statistically significant difference between the parameters (ANCOVA: df = 1, F = 0.20, p = 0.651). The “b” value was found to be statistically different from “3” (p < 0.05) both in two sexes and in all population, thereby it was found that the growth type was “negative allometric” in males, females and all population.

**Figure 4:**
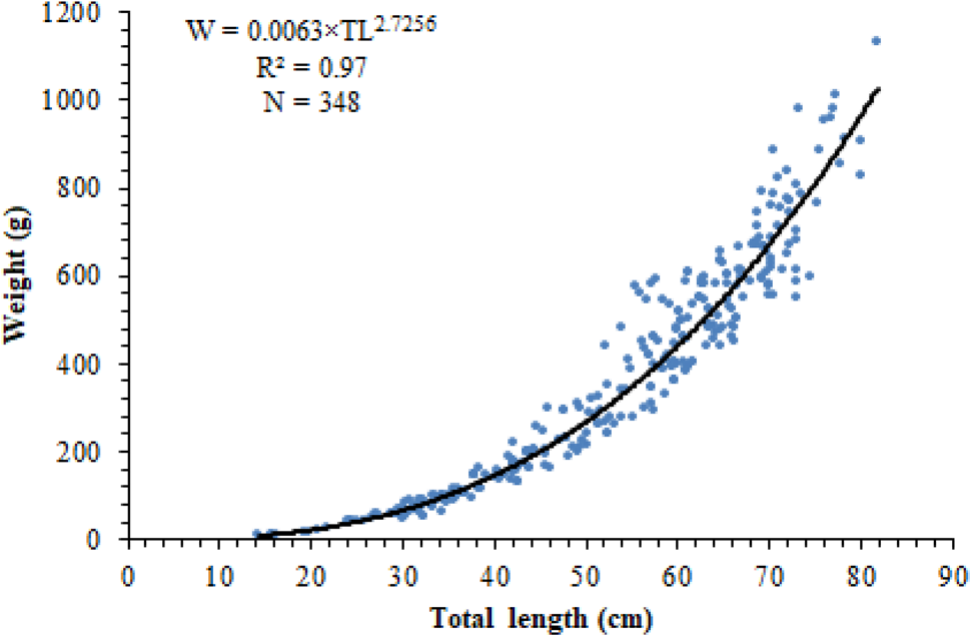
Length–weight relation of *M. mastacembelus* population inhabiting Keban Dam Lake.

The total length–weight relationships were calculated for male, female, and all population as follows:

- W_M_ = 0.0083 × TL^2.6516^ R = 0.97, N = 178, CI_(%95)b_ = 0.065, CI_(%95)a_ = 0.002
- W_F_ = 0.0043 × TL^2.8310^ R = 0.97, N = 170, CI_(%95)b_ = 0.076, CI_(%95)a_ = 0.002
- W_M+F_ = 0.0063 × TL^2.7256^ R = 0.97, N = 348, CI_(%95)b_ = 0.051, CI_(%95)a_ = 0.002 (Fig. 4).

### Condition factor

**Figure 5:**
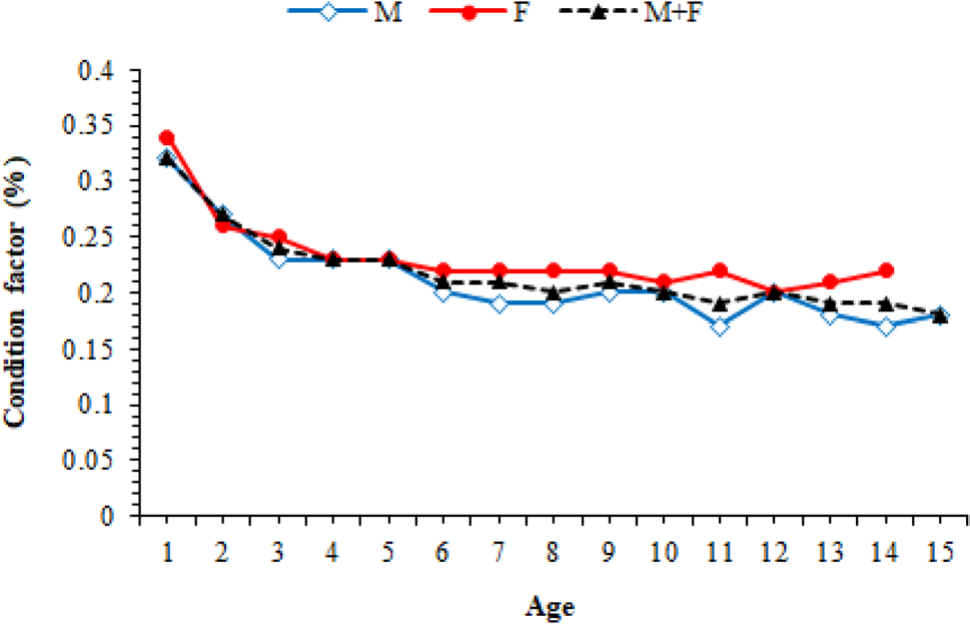
The of condition factor distributions of *M. mastacembelus* population inhabiting Keban Dam Lake.

While the condition factor of males ranged from 0.14 to 0.49, in females reached from 0.15 to 0.35. When the mean values were examined, it was determined that the females had a higher condition than the males and the condition factors decreased with growing age in both sexes, in general (Fig. 5). The difference between the condition factor values of males and females were ascertained statistically different in VI, VII, VIII, XI and XIV age groups (p < 0.05).

### Growth parameters

**Figure 6:**
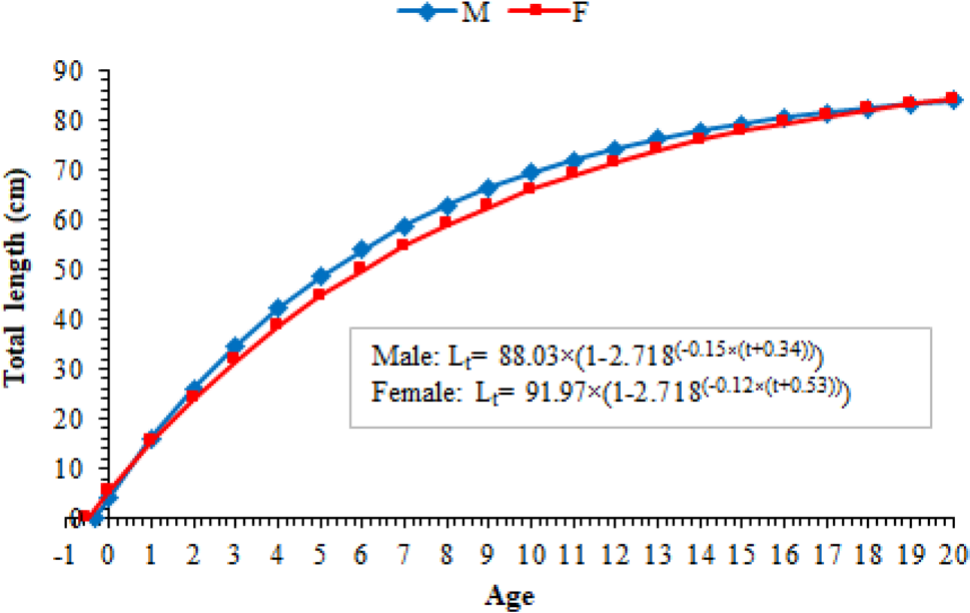
Age-length relations of *M. mastacembelus* population inhabiting Keban Dam Lake, according to sexes.

The von Bertalanffy growth parameters, calculated using age and mean length values, were given in Table 2. It was determined that the differences between measured and estimated (using von Bertalanffy growth parameters) length and weight values were statistically insignificant in both sexes (p > 0.05). When the growth curves of both sexes were investigated, it was seen that the males grew faster than the females in between IV and X age groups, but the difference between in growth rates gradually decreased in the later age groups, and both sexes grew almost at the same rate (Fig. 6). According to the covariance analysis (ANCOVA), it was determined that the growth parameters of the sexes were not statistically different (ANCOVA: df = 1, F = 0.640, p = 0.428).

**Table 2:** VBGE parameters of *M. mastacembelus* population inhabiting Keban Dam Lake, according to sexes.

Parameters	Male ± CI_(%95)_	Female ± CI_(%95)_	Male + female ± CI_(%95)_
*L∞*	88.03 ± 6.17	91.97 ± 6.85	90.99 ± 6.47
*K*	0.15 ± 0.04	0.12 ± 0.02	0.13 ± 0.02
*to*	− 0.34 ± 0.45	− 0.53 ± 0.37	− 0.45 ± 0.41
*W∞*	1189.76	1557.94	1376.49
*Ǿ*	3.065	3.006	2.997

### Mortality and exploitation rates

**Figure 7:**
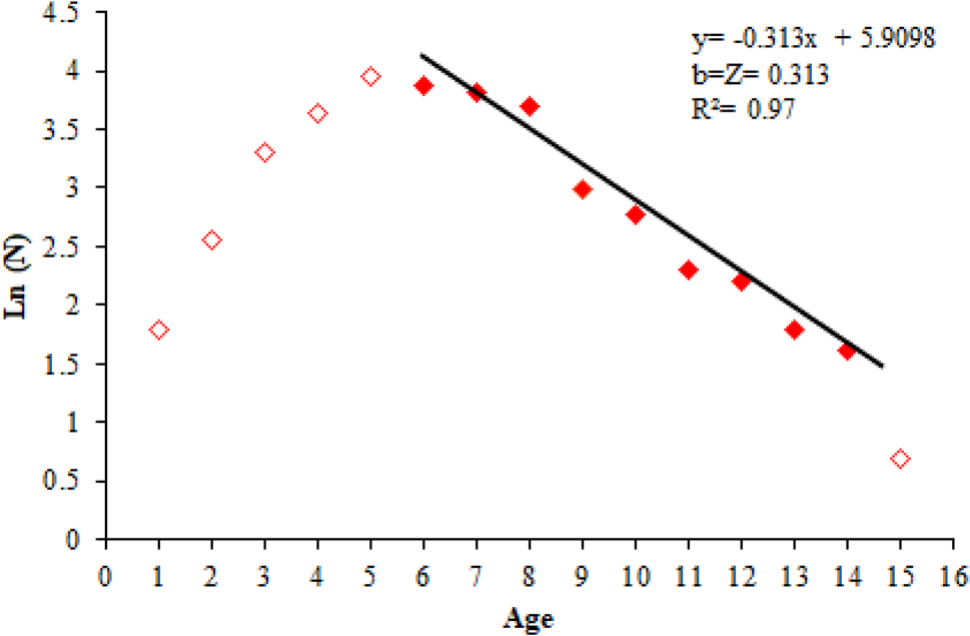
Length converted catch curve.

The natural mortality rate was estimated as M = 0.270 year^−1^ by using Pauly's empirical formula, the total mortality rate was computed as Z = 0.313 year^−1^ by using the length converted catch curve (Fig. 7) and the fishing mortality rate was calculated as F = 0.043 year^−1^ by using the formula F = Z − M. The exploitation rate was computed as 0.137 by using E = F/Z formula. The calculated exploitation rate value is significantly lower than the optimum exploitation rate value, which is assumed to be 0.50.

### Length at first capture (Lc) and recruitment (Lr)

**Figure 8:**
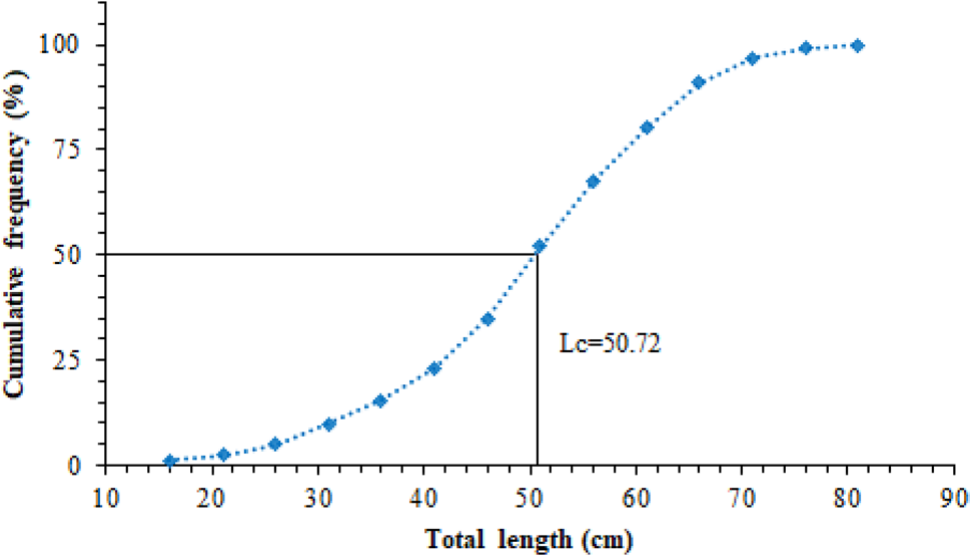
Cumulative length frequency distribution and the length at first capture of *M. mastacembelus* population inhabiting Keban Dam Lake.

The cumulative length-frequency distribution graph was drawn to determine the length at first capture (Lc) of *M. mastacembelus* caught between 2014–2018 in Keban Dam Lake. According to Fig. 8, the length at first capture (Lc) was found to be 50.72 cm, 15.5 cm, which is the midpoint of the smallest size class, was accepted as the length at recruitment (Lr). The ages corresponding to Lc and Lr were calculated as t_c_ = 5.82 year and t_r_ = 0.98 year, respectively, using the VBGE parameters.

### Yield per recruit

**Figure 9:**
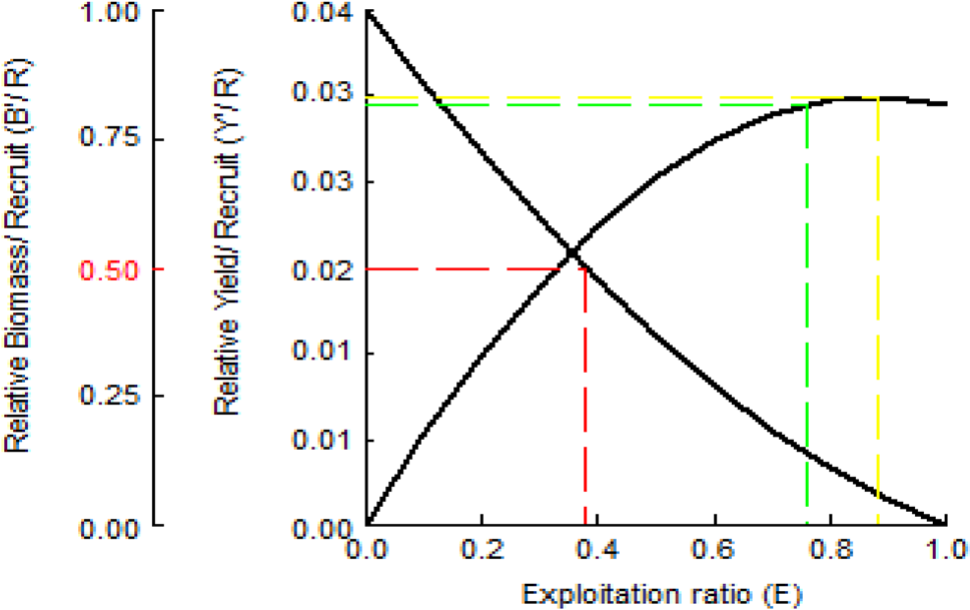
Yield per recruit analysis of *M. mastacembelus* population inhabiting Keban Dam Lake.

The relative yield per recruit model (Y/R) was calculated using the knife-edge method of Beverton and Holt. According to the results, the optimum sustainable yield (E_0.5_) was found to be 0.278, the maximum sustainable yield (E_max_) to be 0.776 and the economic yield (E_0.1_) to be 0.355 (Fig. 9). It was seen that the current exploitation rate calculated as 0.137 is lower than the optimum, maximum and economic yield indices.

## Discussion

In this study; a total of 348 *M. mastacembelus* individuals (178 male and 170 female) were examined and the age distributions were found to vary between 1–15 for males (14.20–81.80 cm) and 1–14 for females (15.60–77.30 cm). Kılıç, ascertained age distribution 1–7 in males (21.0–70.0 cm) and 1–5 in females (25.0–62.0 cm) from Karakaya Dam Lake and two rivers flowed to it. He also reported larger fish specimens from the dam reservoir^28^. In another study conducted in Karakaya Dam Lake, Eroğlu and Şen notified that the age distributions were as 1–9 for males (23.7–80.6 cm) and as 1–8 for females (26.6–68.5 cm)^16^. On the other hand; Pazira et al., from two rivers in southern Iran, reported age distributions as 0–6 for both males (9.5–43.2 cm) and females (4.2–42.5 cm)^29^. In two studies, executed in Ataturk Dam Lake, the age distributions were stated as 1–18 in males (7.0–82.0 cm) and as 1–9 in females (29.0–69.0 cm) by Oymak et al.^17^, and were notified as 1–21 for males (14.4–76.9 cm) and as 1–9 for females (14.9–57.3 cm) by Gümüş et al.^18^. In all of the mentioned studies, since the catching methods of the fish samples are similar (gill nets, fish traps and fishing baskets), it is thought that the differences between fish sizes are due to the difference in living areas instead of the catching technique. In particular, larger Mesopotamian spiny eels samples were caught from lentic systems. It was thought that the inconsistencies between the age data of Kılıç’s^28^ and Eroğlu and Şen’s^16^ studies and the age data of this study were substantially resulted from age reading and validating errors (especially in old fishes).

In this study; “b” value in the length–weight relationship was determined as 2.7256 for all population (M: 2.6516, F: 2.8310). It was found that “b” value statistically different from “3” in both sexes and in all population, and growth types were revealed as negative allometric. “b” value was stated as 1.923 for combined sexes by Kılıç^28^; as 2.524 in males, as 2.144 in females and as 2.275 in all population by Pazira et al.^29^; as 2.43 for males and as 2.95 for females by Oymak et al.^17^; as 2.996 in males, as 2.792 in females and as 2.835 in all individuals by Gümüş et al.^18^. The growth type was reported as isometric only for males in the study of Gümüş et al.^18^. In all other studies, the growth type was notified as negative allometric consistently with our study. This finding is also consistent with the morphological structure of *M. mastacembelus*.

It was estimated that the condition factor values ranged between 0.14–0.49 in males and 0.15–0.35 in females and it decreased as age progress. Pazira et al., reported as 0.16–0.39 in males and as 0.16–0.46 in females in two different rivers in Iran^29^. Eroğlu and Şen stated that the condition factor values varied from 0.17 to 0.30 for males and from 0.19 to 0.27 for females in Karakaya Dam Lake^16^. We think that the differences between the condition factor values resulted from that Eroğlu and Şen’s^16^ findings were average values and the samples used in Pazira et al.’s^29^ study is only obtained from rivers. Because the condition factor values may vary dependently many factors such as age, fish species, habitat, water flow, nutrient, reproductive activity and sampling time^30^.

In this study; L∞ values were identified for males, females and all population as 88.03 (K: 0.15 year^−1^), 91.97 (K: 0.12 year^−1^) and 90.99 (K: 0.13 year^−1^), respectively. It is thought that the differences with other studies in Table 3 are due to disparities in study areas, in age distributions and calculation methods. However, it was determined using the Phi prime test whether the VBGE parameters in the other studies were statistically significant, and it was found that the findings obtained from this study were not statistically different from the findings of Pazira et al.^29^, Oymak et al.^17^ and Gümüş et al.^18^ (for males t = − 3.314, df = 2, p > 0.05; for females t = − 0.792, df = 2, p > 0.05).

**Table 3:** VBGE parameters of different *M. mastacembelus* populations.

References	Sexes	*L∞*	*K*	*to*	*W∞*	*Ǿ*
Kılıç (2002)	M + F	98.59	0.16	− 1.41	1098.92	3.192
Pazira et al. (2005)	M	92.3	0.08	− 1.46	–	2.833
	F	87.3	0.08	− 1.49	–	2.785
Oymak et al. (2009)	M	99.2	0.10	− 0.12	1619.79	2.993
	F	69.2	0.26	− 0.35	777.52	3.095
Gümüş et al. (2010)	M	80.0	0.14	− 0.45	1106.83	2.952
	F	83.6	0.12	− 0.62	1256.59	2.924
	M + F	81.7	0.13	− 0.57	1160.39	2.938
This study	M	88.03	0.15	− 0.34	1189.76	3.065
	F	91.97	0.12	− 0.53	1557.94	3.006
	M + F	90.99	0.13	− 0.45	1376.49	2.997

Natural mortality rate was calculated as M = 0.270, total mortality rate as Z = 0.313, fishing mortality rate as F = 0.043 and exploitation rate as E = 0.137. The exploitation rate is lower than the optimum value that is assumed as 0.50. The optimum, maximum and economic yield indices was calculated as E_0.5_ = 0.278; E_max_ = 0.776; E_0.1_ = 0.355, and the current exploitation rate (E = 0.137) was lower than these values. *M. mastacembelus* is not a direct target species of fishermen in the region, because its long and thin body structure reduces the success of catching this species and because of its snake-like appearance, it is a species not preferred by consumers. So it is expected that the fishing mortality rate and the exploitation rate to be low.

In conclusion, *M. mastacembelus* that is a long-lived and slow-growing species have a low exploitation rate and fishing mortality rate in Keban Dam Lake. In the future, it is thought that the stock estimation studies will be beneficial to learn the economic value of this species.

## References

[1] Eschmeyer, W. N. & Fong, J. D. Species of fish by family/subfamily. https://researcharchive.calacademy.org/research/ichthyology/catalog/SpeciesByFamily.asp#Mastacembelidae [Accessed 10 February 2021] (2021).

[2] Johnson GD, Patterson C (1993). Percomorph phylogeny: A survey of Acanthomorphs and a new proposal. Bull. Mar. Sci..

[3] Britz R, Kottelat M (2003). Descriptive osteology of the family Chaudhuriidae (Teleostei, Synbranchiformes, Mastacembeloidei), with a discussion of its relationships. Am. Mus. Novit..

[4] Brown KJ, Britz R, Bills R, Rüber L, Day JJ (2011). Pectoral fin loss in the Mastacembelidae: A new species from Lake Tanganyika. J. Zool..

[5] Kara C, Güneş H, Gürlek ME, Alp A (2014). Adıyaman bölgesi akarsularında dikenli yılan balığı (*Mastacembelus mastacembelus* Banks & Solander 1794)’nın dağılımı ve bazı morfometrik özellikleri. AquaSt..

[6] Dağlı M, Erdemli AÜ (2009). An investigation on the fish fauna of Balıksuyu Stream (Kilis, Turkey). Int. J. Nat. Eng. Sci..

[7] Geldiay, R. & Balık, S. *Türkiye Tatlısu Balıkları*. VI. Baskı, Ege Üniversitesi Su Ürünleri Yayınları, (Ege Üniversitesi Basımevi, Bornova-Izmir, 2009) **(in Turkish)**.

[8] Vreven EJ, Teugels GG (2005). Redescription of *Mastacembelus liberiensis* Baulenger, 1898 and description of a new West African spiny-eel (Synbranchiformes: Mastacembelidae) from the Konkoure River basin, Guinea. J. Fish Biol..

[9] Jalali B, Barzegar M, Nezamabadi H (2008). Parasitic fauna of the spiny eel, *Mastacembelus mastacembelus* Banks et Solander (Teleostei: Mastacembelidae) in Iran. Iran. J. Vet. Res..

[10] Çakmak, E. Dikenli yılan balığı (*Mastacembelus mastacembelus*)’nın morfolojik ve moleküler özelliklerinin belirlenmesi. Kahramanmaraş Sütçü İmam Üniversitesi, Fen Bilimleri Enstitüsü, Master Thesis, (Kahramanmaraş, 2008) **(in Turkish)**.

[11] Çakmak E, Alp A (2010). Morphological differences among the Mesopotamian spiny eel, *Mastacembelus mastacembelus* (Banks & Solander 1794), populations. Turk. J. Fish. Aquat. Sci..

[12] Şahinöz E, Doğu Z, Aral F (2006). Development of embryos in *Mastacembelus mastacembelus* (Bank & Solender, 1794) (Mesopotamian spiny eel) (Mastacembelidae). Aquac. Res..

[13] Pala G, Tellioğlu A, Eroğlu M, Şen D (2010). The digestive system content of *Mastacembelus mastacembelus* (Banks & Solander, 1794) inhabiting in Karakaya Dam Lake (Malatya-Turkey). Turk. J. Fish. Aquat. Sci..

[14] Eroğlu M, Şen D (2009). Otolith size-total length relationship in spiny eel, *Mastacembelus mastacembelus* (Banks & Solander, 1794) inhabiting in Karakaya Dam Lake (Malatya, Turkey). J. FisheriesSciences.com.

[15] Eroğlu M, Şen D (2012). Relationships between fish age and otolith size in spiny eel: *Mastacembelus mastacembelus* (Banks & Solander, 1794). Bitlis Eren Univ. J. Sci. Technol..

[16] Eroğlu M, Şen D (2007). Reproduction biology of *Mastacembelus simack* (Walbaum, 1792) inhabiting Karakaya Dam Lake (Malatya, Turkey). Int. J. Nat. Eng. Sci..

[17] Oymak SA, Kırankaya ŞG, Doğan N (2009). Growth and reproduction of Mesopotamian spiny eel (*Mastacembelus mastacembelus* Banks and Solander, 1794) in Ataturk Dam Lake (Şanlıurfa), Turkey. J. Appl. Ichthyol..

[18] Gümüş A, Şahinöz E, Doğu Z, Polat N (2010). Age and growth of the Mesopotamian spiny eel, *Mastacembelus mastacembelus* (Banks & Solender, 1794), from southeastern Anatolia. Turk. Zool. Derg..

[19] Anonymous. *Keban Baraj Gölü limnoloji raporu*. DSİ 9. Bölge Müdürlüğü, Su Ürünleri Başmühendisliği. (Keban-Elazığ, 1994) **(in Turkish)**.

[20] Yüksel F, Demirol F, Gündüz F (2013). Leslie population estimation for Turkish crayfish (*Astacus leptodactylus* Esch., 1823) in the Keban Dam Lake, Turkey. Turk. J. Fish. Aquat. Sci..

[21] Google Maps. https://www.google.com/maps/@38.8025012,38.9170508,9z [Accessed 10 February 2021] (2021).

[22] Lagler, K. F., Bardach, J. E., Miller, R. R. & Passino, D. R. M. *Ichthyology* (Wiley, 1977).

[23] Zar, J. H. *Biostatistical Analysis* 4th edn. (Prentice-Hall, 1999).

[24] Pauly, D. *Some Simple Methods for the Assessment of Tropical Fish Stocks* (FAO, 1984).

[25] Sparre, P. & Venema, S. C. *Introduction to Tropical Fish Stock Assessment*. FAO Fisheries Technical Paper, 306/1, Rev. 2, (Rome, 1998).

[26] Munro JL, Pauly D (1983). A simple method for comparing the growth of fishes and invertebrates. FishByte.

[27] Gayanilo, F. C., Sparre, P. & Pauly, D. *FAO-ICLARM Stock Assessment Tools II (FiSAT II). User’s Guide.* FAO Computerized Information Series (Fisheries). No. 8, Revised version, (FAO, Rome, 2005).

[28] Kılıç, H. M. Sultansuyu Deresi, Beyler Deresi ve Karakaya Barajı’nda yaşayan dikenli yılanbalığı (*Mastacembelus simack*)’nın biyoloik özelliklerinin incelenmesi. Osmangazi Universitesi, Fen Bilimleri Enstitüsü, Master Thesis, (Eskişehir, 2002) **(in Turkish)**.

[29] Pazira A, Abdoli A, Kouhgardi E, Yousefifard P (2005). Age structure and growth of the Mesopotamian spiny eel, *Mastacembelus mastacembelus* (Banks & Solander in Russell, 1974) (Mastacembelidae), in southern Iran. Zool. Middle East.

[30] Korkut AY, Kop A, Demirtaş N, Cihaner A (2007). Balık beslemede gelişim performansının izlenme yöntemleri. EgeJFAS.

